# Editorial: Novel delivery systems of flavors and essential oils: exploring potential health applications in foods

**DOI:** 10.3389/fnut.2023.1183878

**Published:** 2023-04-18

**Authors:** Andrew J. Gravelle, Shima Saffarionpour, Amanda Joyce Wright

**Affiliations:** ^1^Department of Food Science and Technology, College of Agricultural and Environmental Sciences, University of California, Davis, Davis, CA, United States; ^2^Department of Chemical Engineering & Applied Chemistry, Faculty of Applied Science & Engineering, University of Toronto, Toronto, ON, Canada; ^3^Department of Human Health and Nutritional Sciences, College of Biological Science, University of Guelph, Guelph, ON, Canada

**Keywords:** encapsulation, flavors, essential oils, foods, delivery

Encapsulation serves to protect and promote the delivery of volatile and otherwise labile compounds for a myriad of applications. It is applied extensively across a wide range of fields, including the pharmaceutical, cosmetics, and food industries. Food applications include the delivery of bioactive nutraceuticals (e.g., vitamins, minerals, pre- and probiotics), as ingredients for food fortification, to enhance or control the release of encapsulated molecules, and to carry flavor and aroma compounds. As carriers of flavors and essential oils, encapsulated systems can contribute to the sensory attributes and palatability of foods and food ingredients, as well as to the biological activities of the encapsulated compounds. As such, interest in exploring novel encapsulation strategies for these applications remains high. This Research Topic aims to highlight recent developments in this area.

The review by Lazar et al. focuses attention on the relevance of encapsulation to gut microbiota and its modulation by essential oils and nanoparticles. It contributes to an enhanced understanding of how nanomaterials, essential oils, and active terpene molecules in encapsulated systems can influence and modulate the human gut microbiota. It discusses the impacts on improving lipolysis, supressing body weight, reducing systemic inflammation, and potential anti-pathogenic activity in the gut. Due to the complexity of the gut microbiome, the impacts of both encapsulated and encapsulant materials are areas requiring further investigation. Indeed, there is significant potential to leverage a better understanding of encapsulation technologies on bioactive delivery, and ultimately health consequences, as mediated through the gut microbiota.

The review by English et al. sheds light on the application of different approaches and techniques for encapsulating flavor compounds. It highlights the influence of preparation technique on particle size and morphology of the produced nano- or micro-particles as encapsulants or colloidal emulsion systems. As key factors influencing the stability of encapsulated flavors, the impact of wall material selection and the interaction of flavors with the encapsulant are discussed. In conclusion, they elaborate on the application of encapsulated flavors in functional foods and ultimately emphasize the need to further explore the influence of encapsulation technique on binding, oxidative stability, and controlled release of flavors.

In their original research contribution, Chen et al. report on the value-added proposition of extracting essential oil from litchi tree flowers which are pruned as a standard cultivation practice for increasing litchi fruit yields. This work highlights the effects of the essential oil on energy intake, metabolic rate, and lipid metabolism under different feeding conditions using nematodes (*Caenorhabditis elegans*) as a biological model. The authors provide evidence that litchi flower essential oil could alleviate lipid accumulation and identified the relevant signaling pathways responsible for this outcome. The work sheds light on the biological mechanism responsible for possible lipid-lowering effects of litchi flower essential oil. It justifies further investigation to extrapolate these findings to other animal models, and ultimately human populations.

Lastly, Tang et al. report on an *in-situ* complex coacervation spray drying process to protect limonene from citrus peel oil in alginate-gelatin microparticles. The researchers included a food-grade latex polymer (ethylcellulose), aiming to improve volatile retention, and characterized the moisture and oxygen barrier properties of the microparticles with the embedded latex excipient. The reported experiments elucidate the influence of latex on microcapsule size and retention of limonene during spray drying. In addition, the impact of storage conditions and relative humidity on the shelf-life and retention of the volatile flavor were explored. Ultimately, limonene encapsulated by complex coacervation with latex ethylcellulose accelerated volatile losses, generating important insights about the nuances of controlled release.

The contributions in this Research Topic showcase that research on delivery systems for flavors and essential oils, and the biological role of both the encapsulants and encapsulated compounds, continue to be an active and exciting area. Encapsulation technologies are central to realizing the potential health benefits of many bioactive molecules. Continued progress will yield further insights, enabling the design of novel and effective delivery strategies that are tailored for a broad range of applications.

## Author contributions

All authors listed have made a substantial, direct, and intellectual contribution to the work and approved it for publication.

